# The Masquerade of Myocardial Infarction as Gastroenteritis: A Diagnostic Challenge

**DOI:** 10.7759/cureus.58441

**Published:** 2024-04-17

**Authors:** Etaro Hashimoto, Kazuya Nagasaki

**Affiliations:** 1 Department of Internal Medicine, Mito Kyodo General Hospital, University of Tsukuba, Mito, JPN

**Keywords:** stemi, acute coronary syndrome, masquerade, gastroenteritis, myocardial infarction

## Abstract

Acute coronary syndrome (ACS) can present with varied symptomatology, often deviating from classic presentations, particularly in patients without the characteristic chest pain. This case report describes an ST-elevation myocardial infarction (STEMI) that closely mimicked acute gastroenteritis, illustrating the challenges of differential diagnosis in atypical ACS presentations. We present the case of a 65-year-old Japanese male with a history of hypertension and dyslipidemia who arrived at the emergency department with acute abdominal pain, vomiting, diarrhea, and fever, symptoms suggesting viral gastroenteritis. The absence of chest pain diverted initial clinical suspicion away from cardiac causes. However, cardiovascular risk factors and a gallop rhythm prompted further cardiovascular evaluation. Subsequent blood tests and electrocardiogram findings suggested an acute myocardial infarction, later confirmed by coronary angiography as STEMI due to a 90% stenosis in the right coronary artery, which was successfully treated with percutaneous coronary intervention. The presentation of myocardial infarction can vary, with non-chest pain symptoms such as vomiting and fever occasionally leading the clinical picture, which may result in diagnostic delays and worsened prognosis. This case was particularly challenging due to the presence of all four symptoms typically associated with gastroenteritis, as well as the sequence of symptom onset being atypical for gastrointestinal diseases. This case exemplifies the need for a high degree of clinical suspicion for ACS in patients with atypical presentations, such as those mimicking gastroenteritis, to prevent misdiagnosis and ensure prompt and appropriate management, especially in patients with known cardiovascular risk factors.

## Introduction

Acute coronary syndrome (ACS) can manifest a spectrum of clinical presentations, not all of which prominently feature chest pain. Atypical presentations are particularly prevalent among older individuals, patients with diabetes, and women. In this case report, we detail the clinical journey of a 65-year-old Japanese male who presented to the emergency department with symptoms emblematic of acute gastroenteritis, namely, severe abdominal pain, vomiting, diarrhea, and fever. Such symptomatology does not typically raise immediate suspicion for myocardial infarction (MI), posing a substantial risk for misdiagnosis. The patient’s presentation was initially not suggestive of acute myocardial infarction (AMI), potentially leading to diagnostic overshadowing and therapeutic delays. This case underscores the critical importance of not overlooking a diagnosis of AMI in the absence of chest pain and elucidates the complexities involved in the clinical assessment of patients with MI presenting without the hallmark symptom. Through this report, we aim to heighten awareness of the necessity for a comprehensive and vigilant approach in the evaluation of atypical presentations of ACS.

## Case presentation

A 65-year-old Japanese male presented to our emergency department in the evening with complaints of abdominal pain, vomiting, and diarrhea. He experienced acute severe umbilical pain with cold sweats at 10 a.m. on the day of the presentation. The pain, which was persistent and not intermittent, was rated 10 on the Numeric Rating Scale. This was followed by an episode of loose stools and four episodes of vomiting. However, the patient did not experience pain outside of the abdominal region. Due to the persistent abdominal pain, he visited our hospital at 6 p.m. After arriving at the hospital, the patient presented with a second episode of loose stools. He reported no consumption of raw or undercooked foods in the preceding week. He lived with his wife and child, neither of whom exhibited similar symptoms. His medical history included hypertension and dyslipidemia, which had been managed through dietary advice alone. However, this approach provided insufficient management of his conditions. He had no history of smoking or alcohol consumption. Upon presentation, his general appearance was good. Vital signs were as follows: temperature, 38.4°C; blood pressure, 149/102 mmHg; heart rate, 92 beats per minute; respiratory rate, 20 breaths per minute; and SpO_2_, 99% on ambient air. Moreover, there were no signs of conjunctival injection or jaundice, and no evidence of pharyngeal erythema or cervical lymphadenopathy was noted. A gallop rhythm was observed in the heart sounds, while pulmonary auscultations were normal. The abdomen was flat and soft with normal bowel sounds. Tenderness was noted from the upper abdomen to the right upper quadrant, with a positive Murphy's sign. No peripheral coldness or lower leg edema was present. Abdominal ultrasound raised suspicion of mild gallbladder enlargement; however, no thickening of the gallbladder wall was present, and no gallstones or sludge were observed. All other abdominal ultrasound findings were normal.

**Figure 1:**
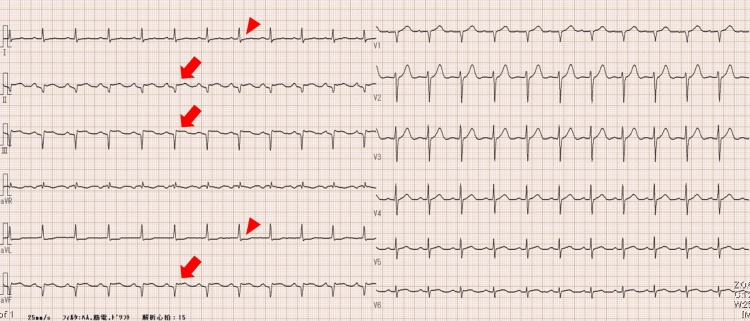
Electrocardiogram. Abnormal Q waves and ST elevation in leads II, III, and aVF (arrow), with ST depression in leads I and aVL (arrowhead).

The above presentation of the patient’s symptoms in the emergency room initially raised the suspicion of viral gastroenteritis. Acute cholecystitis was also considered in the differential diagnosis, supported by a positive Murphy’s sign and findings on abdominal ultrasound. The onset of symptoms accompanied by abdominal pain was deemed atypical for viral gastroenteritis, and the positive Murphy’s sign did not align with viral gastroenteritis. Moreover, diarrhea is not typically associated with cholecystitis. In the differential diagnosis, AMI was considered due to cardiovascular risk factors such as hypertension, dyslipidemia, and male gender. The presence of a gallop rhythm further contributed to the consideration of AMI. An immediate electrocardiogram (ECG) was desired; however, it could not be performed promptly owing to the department’s engagement with other critically ill patients. Blood tests revealed elevated levels, including a white blood cell count of 10,400/μL, creatine phosphokinase 3,111 IU/L, lactate dehydrogenase 583 IU/L, aspartate transaminase 283 IU/L, alanine transaminase 96 IU/L, and mildly elevated biliary enzymes: alkaline phosphatase 89 IU/L and gamma-glutamyl transpeptidase 88 IU/L. Further testing revealed a positive troponin qualitative test, creatine kinase-myocardial band 257 IU/L, and B-type natriuretic peptide 40.4 pg/mL. The ECG indicated abnormal Q waves and ST elevation in leads II, III, and aVF, with ST depression in leads I and aVL (Figure 1).

These findings led to a strong suspicion of ST-elevation myocardial infarction (STEMI), prompting urgent coronary angiography by the cardiologist.

**Figure 2:**
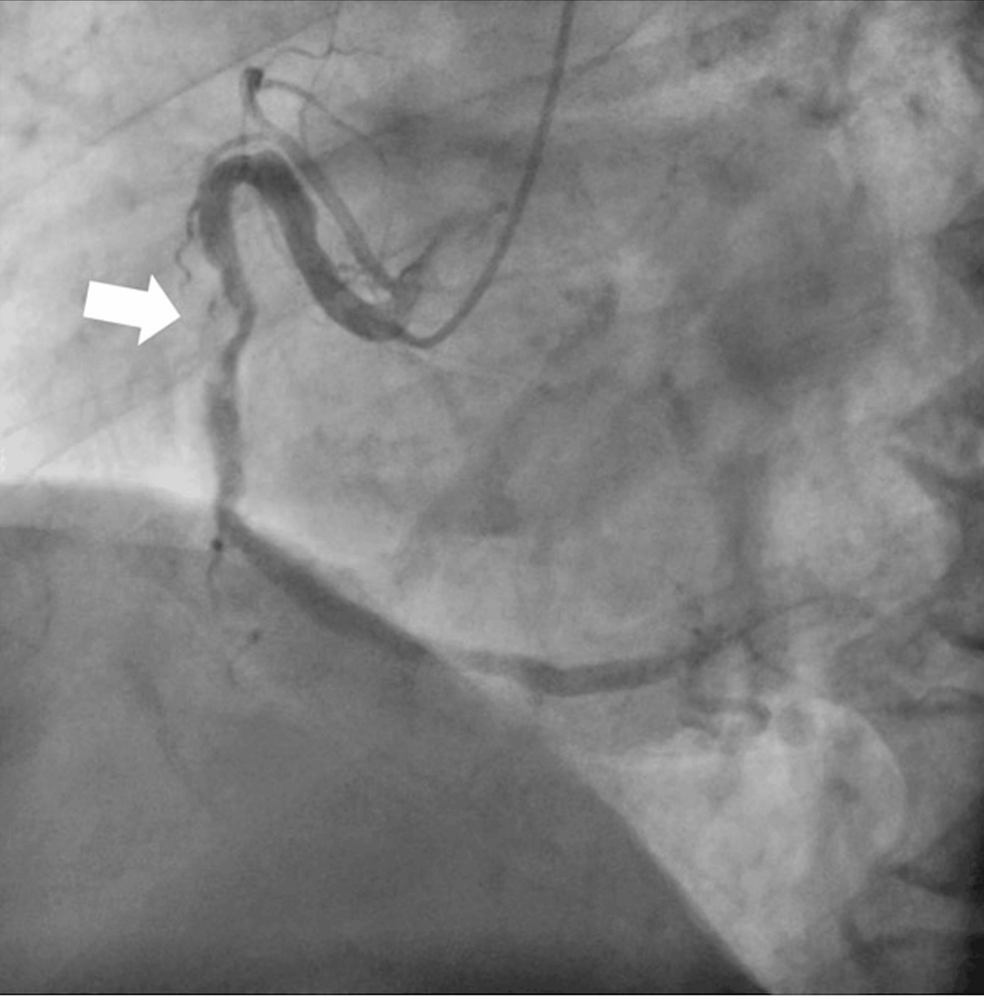
Coronary angiography. Stenosis in the medial segment of the right coronary artery (arrow).

Coronary angiography revealed a 90% stenosis accompanied by plaque rupture in the medial segment of the right coronary artery (RCA) (Figure 2).

Additionally, stenoses of 50% in the proximal segment, 75% in the distal segment of the RCA, and 90% in the proximal segment of the left anterior descending artery (LAD) were identified. No other stenoses or occlusions were observed in the coronary arteries. The stenosis in the medial segment of the RCA was identified as the culprit lesion for the STEMI, and percutaneous coronary intervention (PCI) was performed on this site, followed by admission to the intensive care unit. An abdominal computed tomography scan conducted at this time did not display any gallbladder enlargement. The liver had a maximum craniocaudal dimension of 194 mm, and the spleen had a maximum diameter of 112 mm, indicating hepatomegaly and splenomegaly. After admission, no further episodes of vomiting or diarrhea occurred. The patient became afebrile the day following admission. Considering the progressions of symptoms, the diagnosis was AMI with associated gastrointestinal symptoms and fever. Blood cultures were negative. On the seventh day of hospitalization, PCI was performed on the stenosis in the proximal segment of the LAD. The patient progressed without any complications or emerging issues and was discharged home on the 12th day of hospitalization.

## Discussion

MI presents with a variety of symptoms, including nausea, vomiting, and fever, which may mimic abdominal diseases such as gastroenteritis or gallstone attacks [1-5]. Studies have reported that 8.4-33% of MI cases occur without chest pain, and among patients without chest pain, 24.3% experience vomiting [1,2]. Additionally, abdominal pain is observed in 2% of patients with MI, and fever is seen in 25-50% of patients [3,4]. However, diarrhea in MI is a rare occurrence [5]. Moreover, MI without chest pain often requires more time to diagnose compared to cases with chest pain and is associated with a worse prognosis [1]. In this patient, the presence of all four symptoms commonly associated with gastroenteritis, namely, abdominal pain, vomiting, diarrhea, and fever, made it challenging to differentiate MI from abdominal diseases. However, the sequence of symptom appearance was atypical for gastroenteritis. Usually, gastroenteritis starts with nausea or vomiting, followed by abdominal pain and diarrhea [6]. In this patient, abdominal pain appeared at first, followed by vomiting and diarrhea. Although no studies were identified regarding the sequence of gastrointestinal symptoms in MI, distinctive patterns may be present, such as abdominal pain preceding vomiting, similar to the known presentation in appendicitis [7]. However, vomiting in MI has been reported to correlate with the size of the infarct [8], necessitating consideration of MI in patients who present with vomiting. Furthermore, in this case, the recognition of cardiovascular risk factors (hypertension, dyslipidemia, and male gender) and a gallop rhythm, both associated with MI, served as clues [9,10]. Although rare, this case is significant in demonstrating that MI can present with symptoms typical of gastroenteritis and underscores the need to consider MI when the progression of symptoms does not follow a typical course for gastroenteritis.

In this case, the misleading distractors included gastrointestinal symptoms, positive Murphy’s sign, and tenderness from the epigastric region to the right upper quadrant. The positive Murphy’s sign and right upper quadrant tenderness could potentially be attributed to acute hepatic enlargement induced by congestion. This was an inferior wall MI, and systemic congestion would align with the aforementioned diagnosis [11]. The epigastric tenderness could be attributed to hepatic congestion. However, gastritis or gastric ulcers, known complications of MI, could also be contributing factors [12]. As no upper gastrointestinal endoscopy was performed in this case, the presence of a peptic disease was not confirmed.

In the emergency department setting, assessing whether a patient’s symptoms could indicate a potentially fatal illness is imperative. When patients present with abdominal pain, fever, vomiting, and diarrhea, the initial consideration often leans toward acute gastroenteritis, a highly prevalent condition. However, even when all symptoms suggest gastroenteritis, it is crucial to consider MI if a definitive diagnosis remains elusive.

## Conclusions

This case report underscores the diversity of STEMI presentations, with a focus on those mimicking acute gastroenteritis, absent the common symptom of chest pain. It highlights the need for clinicians to maintain a broad differential diagnosis when evaluating patients presenting with symptoms reminiscent of gastroenteritis. The rapid identification and management of such atypical cases of STEMI are crucial for favorable outcomes. This report serves as a pivotal reminder of the importance of considering coronary etiologies in patients presenting with acute gastrointestinal symptoms, even in the absence of chest pain.
